# Itching of the Mouth

**Published:** 1895-10

**Authors:** 


### Itching of the Mouth.

In the *Deutsch Medizinal-Zeitung* for August 15th there is an abstract of an article entitled Pruritus Oris, by Tommasoli, published in the *Giornale Italiano delle malattie e della pelle*, 1894, No. 3. The author relates the case of a peasant woman, 33 years old, without anything remarkable in her history, who for four years had suffered with an itching and biting sensation in the cavity of the mouth, which compelled her to bite her tongue and to compress the mucous membrane of the cheeks between her teeth. The affection was aggravated in paroxysms, and occasionally she was entirely free from it. The chief situation of the abnormal sensation was in the tongue, which often bled from severe bites. Examination of the cavity of the mouth showed on the mucous membrane of the cheek two whitish, almost horizontal and symmetrical stripes, which were nearly as long as the alveolar processes, to which they corresponded roughly in their course and of which a slight impression was to be seen. Beginning at the last molars, these stripes reached almost to the angles of the mouth. The epithelium on these stripes was moist and soft, but not apparently destroyed. The whole looked like a linear zone of œdematous swollen mucous membrane. Yet, on palpitation, the stripes felt like cords, indolent and not yielding in the slightest to digital compression. All the rest of the mucous membrane, as well as the tongue, appeared sound. The author believes that this was a chronic paroxysmal paræsthesia of the buccal mucous membrane, giving rise to actual changes in those parts of the mucous membrane that were most affected. He gives the name preitus to this affection.

